# A phase I study to assess the mass balance, excretion, and pharmacokinetics of [^14^C]-ixazomib, an oral proteasome inhibitor, in patients with advanced solid tumors

**DOI:** 10.1007/s10637-017-0509-1

**Published:** 2017-09-21

**Authors:** Neeraj Gupta, Steven Zhang, Sandeepraj Pusalkar, Mihaela Plesescu, Swapan Chowdhury, Michael J. Hanley, Bingxia Wang, Cindy Xia, Xiaoquan Zhang, Karthik Venkatakrishnan, Dale R. Shepard

**Affiliations:** 1 Millennium Pharmaceuticals, Inc., a wholly owned subsidiary of Takeda Pharmaceutical Company Limited, 40 Landsdowne Street, Cambridge, MA 02139 USA; 2 Cleveland Clinic Taussig Cancer Institute, Cleveland, OH USA

**Keywords:** Ixazomib, Pharmacokinetics, Mass balance, Total radioactivity, ADME, AMS

## Abstract

This two-part, phase I study evaluated the mass balance, excretion, pharmacokinetics (PK), and safety of ixazomib in patients with advanced solid tumors. In Part A of the study, patients received a single 4.1 mg oral solution dose of [^14^C]-ixazomib containing ~500 nCi total radioactivity (TRA), followed by non-radiolabeled ixazomib (4 mg capsule) on days 14 and 21 of the 35-day PK cycle. Patients were confined to the clinic for the first 168 h post dose and returned for 24 h overnight clinic visits on days 14, 21, 28, and 35. Blood, urine, and fecal samples were collected during Part A to assess the mass balance (by accelerator mass spectrometry), excretion, and PK of ixazomib. During Part B of the study, patients received non-radiolabeled ixazomib (4 mg capsules) on days 1, 8, and 15 of 28-day cycles. After oral administration, ixazomib was rapidly absorbed with a median plasma T_max_ of 0.5 h and represented 70% of total drug-related material in plasma. The mean total recovery of administered TRA was 83.9%; 62.1% in urine and 21.8% in feces. Only 3.23% of the administered dose was recovered in urine as unchanged drug up to 168 h post dose, suggesting that most of the TRA in urine was attributable to metabolites. All patients experienced a treatment-emergent adverse event, which most commonly involved the gastrointestinal system. These findings suggest that ixazomib is extensively metabolized, with urine representing the predominant route of excretion of drug-related material.

Trial ID: ClinicalTrials.gov # NCT01953783.

## Introduction

Ixazomib is a small molecule inhibitor of the 20S proteasome that is administered orally [1]. Ixazomib has been investigated in clinical studies in a broad range of malignancies, with encouraging evidence of activity in patients with multiple myeloma (MM) and systemic light chain (AL) amyloidosis [2–9]. In the phase III TOURMALINE-MM1 trial in patients with relapsed/refractory MM (RRMM), the addition of ixazomib (4 mg starting dose) to lenalidomide and dexamethasone resulted in a significant improvement in progression-free survival (median, 20.6 versus 14.7 months; hazard ratio 0.74, *p* = 0.012), with limited additional toxicity versus lenalidomide and dexamethasone in combination with placebo [7].

Based upon these data, ixazomib was approved in the United States and European Union for use in combination with lenalidomide and dexamethasone for the treatment of patients with MM who have received at least one prior therapy [10], and is now approved in more than 40 countries worldwide [11–13]. The efficacy and safety of ixazomib in MM is being further investigated in multiple ongoing randomized, placebo-controlled, phase III studies in the following settings: newly diagnosed MM (TOURMALINE-MM2; NCT01850524); MM maintenance therapy following autologous stem cell transplantation (ASCT) (TOURMALINE-MM3; NCT02181413); and MM maintenance therapy after initial induction therapy without ASCT (TOURMALINE-MM4; NCT02312258).

After oral administration, ixazomib is rapidly absorbed with a median time to first maximum observed concentration (T_max_) of approximately 1 h post dose [10]. A high-fat meal decreases both the rate and extent of ixazomib absorption [14]. Based on a population pharmacokinetic (PK) analysis of data from 755 patients enrolled across 10 clinical trials, including the global phase III TOURMALINE-MM1 study, the absolute oral bioavailability and steady-state volume of distribution of ixazomib were estimated to be 58% and 543 L, respectively, with a systemic clearance of 1.86 L/h and a terminal disposition phase half-life (t_1/2_) of 9.5 days [15]. Ixazomib is highly bound to plasma proteins (99%), primarily serum albumin [16, 17]. At clinically relevant ixazomib concentrations, in vitro studies indicate that ixazomib is metabolized by multiple cytochrome P450 (CYP) and non-CYP proteins with CYP450 enzymes playing only a minor role. At concentrations exceeding those observed clinically (10 μM), ixazomib was metabolized by multiple CYP isoforms with estimated relative contributions of 3A4 (42%), 1A2 (26%), 2B6 (16%), 2C8 (6%), 2C19 (5%), 2D6 (5%), and 2C9 (<1%) [10].

On the basis of population PK analyses, mild renal impairment (creatinine clearance [CrCl] ≥30 mL/min) and mild hepatic impairment (total bilirubin ≤1.5 x the upper limit of normal [ULN]) had no clinically meaningful effect on ixazomib PK [15]. In dedicated phase I studies, plasma exposures of ixazomib were increased in patients with severe renal impairment (CrCl <30 mL/min), end-stage renal disease requiring dialysis, moderate hepatic impairment (total bilirubin >1.5–3 x ULN), or severe hepatic impairment (total bilirubin >3 x ULN) as compared with patients with normal organ function [16, 17]. Therefore, reduced doses of ixazomib are recommended in these patient populations [10]. Drug-drug interaction studies with the strong CYP3A inhibitors ketoconazole and clarithromycin, and the pleiotropic strong inducer rifampin, demonstrated that strong CYP3A inhibitors have no significant effect on ixazomib PK, while co-administration of rifampin reduced plasma exposures (AUC) of ixazomib by 74% [18].

Mass balance and excretion studies are a standard part of the drug development process and aim to elucidate the metabolic fate of the drug by characterizing the plasma PK and excretion of unchanged drug and metabolites by measuring total radioactivity (TRA) [19]. Accordingly, this phase I, human absorption, distribution, metabolism, and excretion (ADME) study (NCT01953783) was conducted to characterize the mass balance, PK, routes of excretion, and safety of ixazomib when administered as a radiolabeled oral solution.

## Methods

### Overall study design and objectives

This was a two-part, phase I, human ADME study to characterize the mass balance, PK, metabolism, and excretion of oral ixazomib in patients with advanced solid tumors or lymphoma. Part A of the study was considered the period for PK assessments.

The primary objectives of the study were: (1) to assess the mass balance (i.e., cumulative excretion of TRA in urine and feces) of ixazomib following a single oral solution dose of 4.1 mg [^14^C]-ixazomib containing approximately 500 nCi of TRA (specific activity of 125 nCi/mg); and (2) to characterize the PK of ixazomib in plasma and urine, and of TRA in plasma and whole blood, after a single oral solution dose of 4.1 mg [^14^C]-ixazomib. The secondary objective was to assess the safety and tolerability of multiple-dose ixazomib administration in patients with advanced solid tumors or lymphoma.

The study protocol and protocol amendments were approved by the institutional review board at the participating center. All patients provided written informed consent, and the trial was conducted according to the stipulations set out in the Declaration of Helsinki and International Conference on Harmonization Guideline for Good Clinical Practice. The study was registered at www.clinicaltrials.gov as NCT01953783.

### Patients

Patients were eligible to participate in the study if they were aged ≥18 years with histologically or cytologically confirmed metastatic solid tumors or lymphomas for which no standard, curative, or life-prolonging therapies existed. All patients had an Eastern Cooperative Oncology Group performance status of 0, 1, or 2. An absolute neutrophil count ≥2.5 × 10^9^ L, platelet count >100 × 10^9^ mL, total bilirubin <1.5 x ULN, alanine transaminase and aspartate transaminase <2.5 x ULN, and a CrCl ≥30 mL/min were also required for study participation. Eligible patients had to have recovered from the reversible effects of prior anticancer treatment.

Key exclusion criteria for the study included: female patients who were lactating, breastfeeding, or pregnant; patients with grade > 2 peripheral neuropathy; patients with symptomatic brain metastasis; patients with a medical history of urinary or fecal incontinence; radiotherapy or treatment with any investigational products within 21 days before the first dose of ixazomib; and systemic treatment with strong or moderate inhibitors of CYP1A2 or CYP3A, or strong CYP3A inducers, within 14 days before ixazomib administration.

### Treatments

**Fig. 1:**
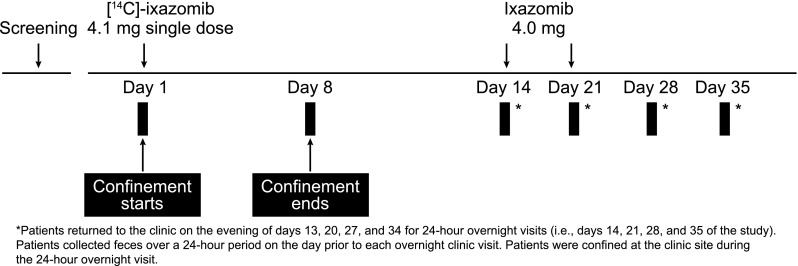
Treatment schema

This study was conducted in two parts: a 35-day PK cycle (Part A), followed by repeated 28-day treatment cycles (Part B; Fig. 1). In Part A of the study, patients who met the inclusion and exclusion criteria were admitted to the study clinic on the morning of day 1 for the initial confinement period (days 1–8). Patients received a single 10 mL oral solution dose of 4.1 mg [^14^C]-ixazomib containing approximately 500 nCi of TRA, followed by 3 × 10 mL water rinses of the dosing cup. Patients then drank approximately 200 mL of water. Patients returned to the clinic on days 14, 21, 28, and 35 for a 24 h overnight clinic visit. Non-radiolabeled doses of ixazomib (4 mg capsule) were administered on days 14 and 21. After completing their day 35 assessments in Part A, patients were permitted to continue into Part B of the study, where they received non-radiolabeled ixazomib (4 mg capsules) once weekly on days 1, 8, and 15 of each 28-day treatment cycle until disease progression or unacceptable toxicity. All doses of ixazomib were administered on an empty stomach, at least 1 h before or at least 2 h after food.

### Assessments

Plasma and whole blood samples were collected at the following time points after ixazomib administration on day 1 of Part A: 0 (predose), 0.5, 1, 2, 3, 4, 8, 24, 48, 72, 96, 120, 144, 168, 312 (day 14), 480 (day 21), 648 (day 28), and 816 (day 35) h post dose. Complete urinary and fecal output was collected continuously during the initial confinement period of Part A (days 1–8). Patients were discharged from the clinic on day 8 and returned on days 14, 21, 28, and 35 for a 24 h overnight visit. Urinary and fecal output was collected during each of these 24 h overnight clinic visits. Patients were also instructed to collect all feces at home for the 24 h period before each overnight clinic visit (days 13, 20, 27, and 34).

### Analysis of ixazomib concentrations in plasma and urine

Plasma and urine concentrations of ixazomib were measured using a validated liquid chromatography/tandem mass spectrometry (LC/MS/MS) assay with a quantitation range of 0.5–500 ng/mL [16].

### Analysis of TRA in blood, plasma, feces, and urine

To achieve enhanced analytical sensitivity, accelerator mass spectrometry (AMS) was used to determine TRA in urine, feces, whole blood, and plasma samples. AMS enabled detection of tracer levels of radioactivity over a long time period post ixazomib administration, therefore enabling outpatient treatment during the study, which greatly reduced the burden on participating patients [20, 21]. At the testing facility (Accium BioSciences, Seattle, WA, USA), samples were assigned a unique tracking number, labeled, and stored at −70 °C until further analysis. Samples were subsequently removed from storage, thawed at room temperature, and thoroughly mixed. A known aliquot of each specimen was transferred to a pre-baked quartz tube and a known amount of carbon carrier added. Samples were then dried using vacuum centrifugation and submitted for graphitization; a known amount of each specimen was combusted and then reduced to graphite for measurement of total ^14^C in the sample [22]. The resulting iron-graphite mixture was pressed into individual cathodes and submitted for AMS measurement on a National Electrostatics 5.2.1.Corporation (NEC) 1.5SDH Compact AMS System (Middleton, WI) running data acquisition software developed by NEC. Total carbon measurements were performed on a Shimadzu V-Series Total 5.2.2.Organic Carbon Analyzer, which uses oxidative combustion combined with non-dispersive infrared detection for the determination of total carbon concentrations. A typical batch contained unknown samples, certified standards to normalize all measurements, machine blanks (^14^C–free graphite of natural origin) to assess the sensitivity of the spectrometer, and chemical blanks (samples prepared with a ^14^C–free substance) to characterize the extraneous carbon introduced during sample preparation. For total carbon measurement, a reference value of 43.7 mg C/mL and 109.8 mg C/mL for plasma and whole blood, respectively, was used [23]. The AMS isotope ratio was converted to ng-eq/mL of whole blood, plasma, urine, or fecal homogenate. The TRA concentrations in urine and feces were further converted to percentage of dose recovered in urine and feces based upon the radioactivity dose administered to each patient.

### PK analyses

PK analyses were based on the PK-evaluable population, which was defined as patients who received the protocol-specified single [^14^C]-ixazomib dose in Part A of the study, did not receive any excluded concomitant medications during Part A, and had sufficient concentration-time data to permit the reliable estimation of PK parameters and mass balance. The plasma PK of ixazomib was characterized over the first 14 days post dose, while the urine PK and excretion of ixazomib was assessed over the first 7 days post dose. TRA in plasma, whole blood, urine, and feces was assessed over the 35-day post-dose period in Part A. Mass balance of radiolabeled ixazomib was characterized based on the sum of cumulative amounts excreted in feces and urine during the continuous collection interval up to 7 days post dose (day 8, 168 h), and the interpolated amounts excreted in feces and urine between the weekly intermittent collections up to 35 days post dose. For the intervals of time during which urine and feces were not collected, the interval recovery of TRA was estimated as the area under the excretion rate-time curve from the end of the preceding collection interval to the start of the subsequent collection interval. PK parameters were calculated by non-compartmental analysis methods (Phoenix WinNonlin version 6.2, Certara, Princeton, NJ, USA).

## Results

### Patients

Seven patients were enrolled in the study and were included in the safety population (Table 1). Five of the seven patients were female and five were white. The median age was 61 years (range, 52–76), and the median weight was 79.8 kg (range, 57.4–101 kg). All patients had solid tumors, with the most common cancer types being breast cancer and cholangiocarcinoma (two patients each). All patients had received prior antineoplastic therapy, two patients had received prior radiation, and three patients had received prior surgery. Five of the seven patients were included in the PK-evaluable population. Two patients were excluded from the PK-evaluable population due to missing samples (one patient) and failing to complete Part A of the study (one patient, due to progressive disease).

**Table 1:** Baseline demographics and disease characteristics (safety population)

Characteristic	All patients (*N* = 7)
Median age, years (range)	61 (52–76)
Male/female, *n* (%)	2 (29) / 5 (71)
Race	
White	5 (71)
Black/African American	1 (14)
Not reported	1 (14)
ECOG performance status 0/1, *n* (%)	2 (29) / 5 (71)
Solid tumor type, *n* (%)	
Bladder	1 (14)
Breast	2 (29)
Cholangiocarcinoma	2 (29)
Ovarian	1 (14)
Pancreatic	1 (14)
Solid tumor disease stage at study enrolment, *n* (%)	
IIIA	1 (14)
IV	2 (29)
Not available	4 (57)
Median time from initial diagnosis to first dose of ixazomib, months (range)	31.5 (7–181)
Received prior radiation, *n* (%)	2 (29)
Received prior surgery, *n* (%)	3 (43)

*ECOG* Eastern Cooperative Oncology Group

The median number of treatment cycles for all 5 treated patients in part B of the study was 5 (range 1-15). The median cumulative ixazomib treatment exposure for all 5 treated patients in part B of the study was 56 mg (range 12–172 mg), administered in a median of 14 doses (range 3–43) over a median of 127 days (range 15–392). Five patients discontinued due to progressive disease (two patients after cycle 1 [Part A], one after cycle 2, one after cycle 4, and one after cycle 5), one patient withdrew from the study (after cycle 5), and one patient discontinued for an unspecified reason (after cycle 15).

### Pharmacokinetics of ixazomib and TRA

**Fig. 2:**
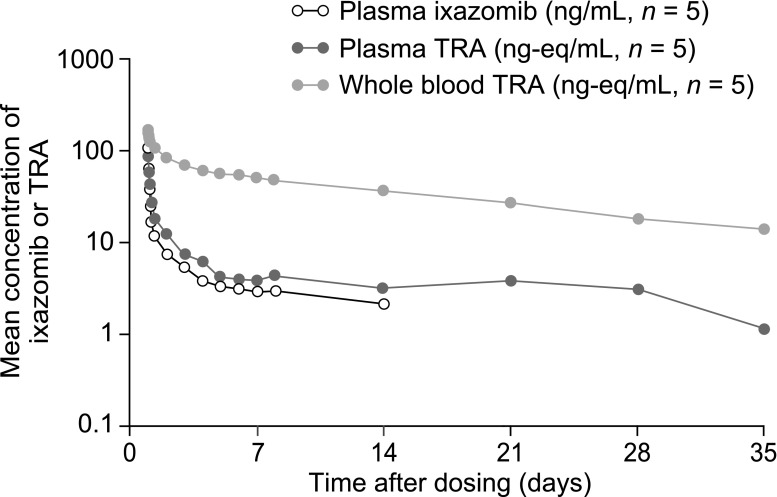
Mean plasma concentration–time profiles for ixazomib and TRA, and whole blood TRA–time profile following administration of a single 4.1 mg oral solution dose of [^14^C]-ixazomib. *TRA* total radioactivity

After administration of a single oral solution dose of 4.1 mg [^14^C]-ixazomib, ixazomib was rapidly absorbed with a median plasma T_max_ of 0.5 h (Table 2; Fig. 2). The geometric mean (coefficient of variation [%CV]) plasma ixazomib maximum observed concentration (C_max_) and AUC_0–312_ (area under the curve from 0 to 312 h) values were 89.1 (62.3) ng/mL and 1180 (46.0) ng*h/mL, respectively. The mean (SD) percent of the administered dose recovered in urine as unchanged drug over 168 h post dose was 3.23 (2.13)%. The geometric mean (%CV) renal clearance was 0.119 (52.0) L/h (Table 2).

**Table 2:** Ixazomib PK parameters in plasma and urine

Parameter	PK-evaluable population (*N* = 5)
Plasma	
Median T_max_, h (range)	0.5 (0.5–0.6)
Geometric mean C_max_, ng/mL (%CV)	89.1 (62.3)
Geometric mean AUC_0–168_, ng*h/mL (%CV)	847 (46.8)
Geometric mean AUC_0–312_, ng*h/mL (%CV)	1180 (46.0)
Urine	
Geometric mean CL_R_, L/h (%CV)	0.119 (52.0)
Mean urinary recovery, % (SD)	3.23 (2.13)

*AUC*_*0–t*_ area under the plasma concentration–time curve from time 0 to time t hours post dose, *CL*_*R*_ renal clearance, *C*_*max*_ maximum observed concentration, *CV* coefficient of variation, *PK* pharmacokinetic, *SD* standard deviation, *T*_*max*_ time to first maximum observed concentration

The median T_max_ for TRA was 0.5 h in plasma and 0.6 h in whole blood (Table 3). Plasma concentrations of TRA were higher than those of ixazomib, indicating the presence of ixazomib metabolites in the systemic circulation (Fig. 2). The geometric mean (%CV) plasma AUC_0–312_ for TRA was 1720 (44.0) ng-eq*h/mL (Table 3). Based on the plasma AUC_0–312_ ratio of ixazomib to ixazomib-related TRA, ixazomib accounted for the majority (% mean [SD] 70.0 [14.2] %) of the circulating component in plasma. Concentrations of TRA were higher in whole blood than in plasma, suggesting preferential distribution of ixazomib-related material into blood cells relative to plasma (Table 3). The mean blood TRA AUC_0–312_ to plasma TRA AUC_0–312_ ratio was 10.5. Plasma concentrations of TRA initially declined more rapidly than those in whole blood; however, TRA appeared to be eliminated from both plasma and whole blood at a similar rate based on visual inspection of the terminal disposition phase of the mean semi-logarithmic concentration versus time plots (Fig. 2).

**Table 3:** PK parameters for ixazomib-related TRA in plasma and whole blood (PK-evaluable population)

Parameter	Plasma (*N* = 5)	Whole blood (*N* = 5)
Median T_max_, h (range)	0.5 (0.5–4.0)	0.6 (0.5–2.0)
Geometric mean C_max_, ng-eq/mL (%CV)	78.8 (54.4)	182 (39.1)
Geometric mean AUC_0–168_, ng-eq*h/mL (%CV)	1240 (40.8)	11,200 (19.3)
Geometric mean AUC_0–312_, ng-eq*h/mL (%CV)	1720 (44.0)	17,300 (19.3)
Geometric mean AUC_0–816_, ng-eq*h/mL (%CV)	2980 (56.9)	29,200 (16.0)
Mean blood AUC_0–312_/plasma AUC_0–312_ (SD)	10.5 (3.56)	

*AUC*_*0–t*_ area under the plasma concentration–time curve from time 0 to time t hours post dose, *C*_*max*_ maximum observed concentration, *CV* coefficient of variation, *PK* pharmacokinetic, *SD* standard deviation, *T*_*max*_ time to first maximum observed concentration, *TRA* total radioactivity

### Recovery of TRA in urine and feces

**Fig. 3:**
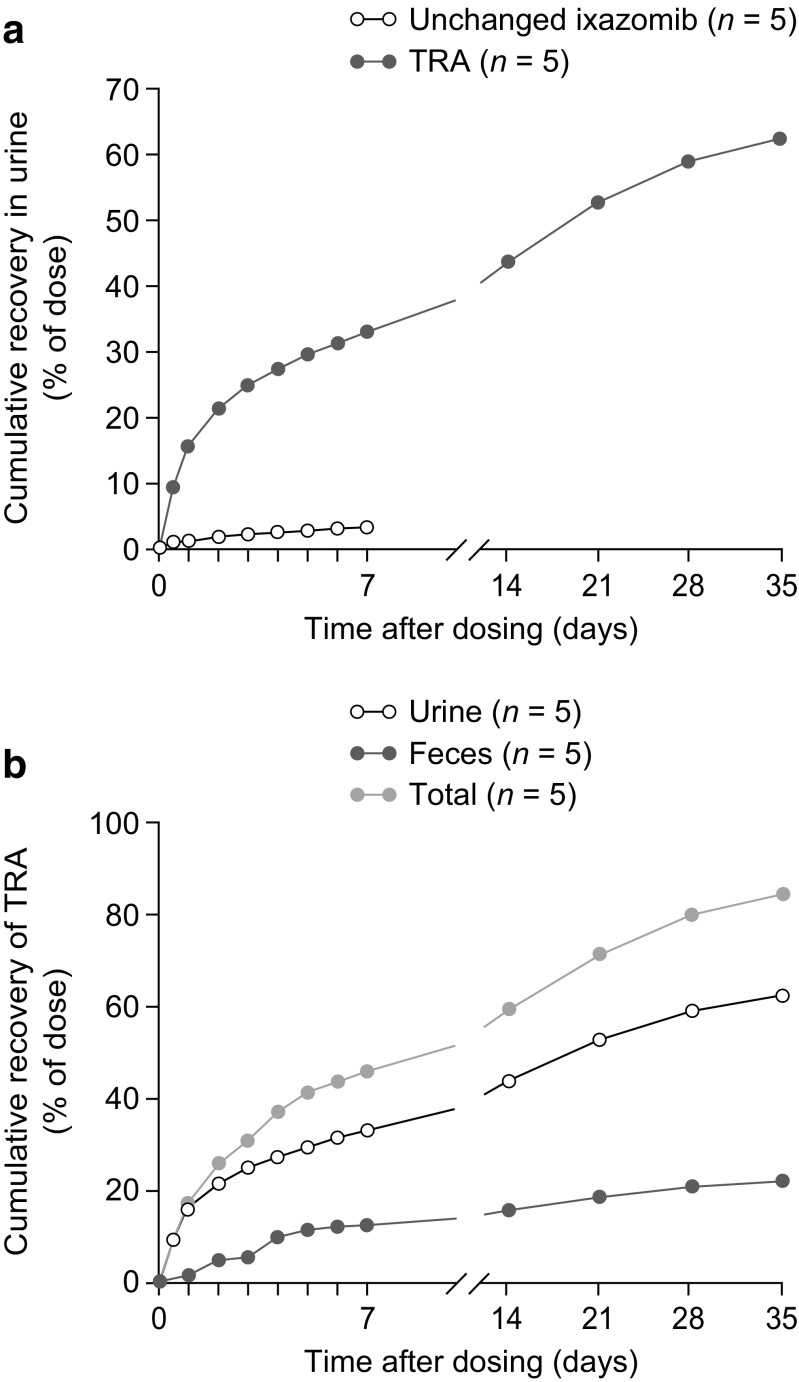
(**a**) Mean percentage cumulative recovery in urine over time for unchanged ixazomib and ixazomib-related TRA, and (**b**) mean percentage cumulative urine, fecal, and total recovery over time for ixazomib-related TRA, following administration of a single oral solution dose of 4.1 mg [^14^C]-ixazomib. *TRA* total radioactivity

The mean (SD) total recovery of the administered TRA dose in urine and feces over the 35-day duration of Part A was 83.9% (20.7; Fig. 3; Table 4). The majority of radioactivity (mean SD 62.1% [21.2]) was recovered in the urine, and 21.8% (3.41) was recovered in feces. Less than 3.5% of the administered dose was recovered in the urine as unchanged drug up to 168 h post dose, suggesting that most of the TRA in urine was attributable to metabolites (Fig. 3a). The mean cumulative excretion of TRA in urine and feces was 59.0% by day 14 post dose. The rate of excretion reached a plateau on day 28 (mean total recovery of 79.4%) with less than a 1% daily increment in excretion thereafter (Fig. 3b).

**Table 4:** Individual cumulative ixazomib-related TRA recovered in urine and feces as a percentage of the administered dose (PK-evaluable population)

Sample	Patient 001	Patient 002	Patient 005	Patient 006	Patient 007	Mean (SD)
Urine (days 1–35, %)	40.8	96.0	58.5	49.3	65.7	62.1 (21.2)
Feces (days 1–35, %)	18.3	18.7	26.4	23.7	21.9	21.8 (3.41)
Total recovery (%)	59.1	115	84.9	73.0	87.6	83.9 (20.7)

*PK* pharmacokinetic, *SD* standard deviation, *TRA* total radioactivity

### Safety

All patients (7/7, 100%) experienced a treatment-emergent adverse event (TEAE), and the majority of patients (6/7, 86%) had a drug-related event. The majority of AEs were mild in severity with only two patients experiencing a grade 3 AE (influenza and platelet count decreased). Overall, the most frequently occurring events included diarrhea (71%), headache (43%), abdominal pain (43%), and decreased appetite (43%). Overall by body system, TEAEs were most commonly reported affecting the gastrointestinal system, with all patients experiencing an event (diarrhea [71%], abdominal pain [43%], nausea, vomiting, and constipation [29% each], dry mouth [14%]). One patient experienced a serious adverse event of influenza; this was deemed unrelated to study drug and resolved. No patients discontinued the study prematurely or required a dose reduction due to an adverse event, and no patients died while on study.

### Efficacy

Five of 7 patients entered part B (cholangiocarcinoma [*n* = 2], bladder [*n* = 1], breast [n = 1], and ovarian cancer [n = 1]). During the study period, 5 patients developed progressive disease. Two patients had long-term stable disease and remained on study for 162 (cholangiocarcinoma) and 427 (ovarian cancer) days, respectively.

## Discussion

This phase I human ADME study assessed the mass balance, routes of excretion, and PK of the oral proteasome inhibitor ixazomib when administered as a [^14^C]-ixazomib oral solution to patients with advanced solid tumors or lymphoma. Due to stability concerns with ^14^C–labeled ixazomib and the long t_1/2_ of ixazomib (geometric mean t_1/2_ of 9.5 days) [10], [^14^C]-ixazomib was administered at low specific activity and at a low radioactive dose (approximately 500 nCi nominal dose). The radioactive dose used in this study was approximately 200-fold lower than the dose used in traditional radiolabeled ADME studies. This necessitated the use of a very sensitive method to accurately determine the recovery of radioactivity in urine, feces, whole blood, and plasma samples. In addition, for drugs with long half-lives, the recovery of the radioactivity dose in mass balance studies is historically low, potentially because of inadequate analytical sensitivity [19]. AMS counts the isotope ratio and measures the actual amount of ^14^C in the sample, resulting in a technique >1000 times more sensitive than liquid scintillation counting (the method typically used in radiolabeled studies that include the administration of a high radioactive dose). In addition, AMS allows for detection of tracer levels of radioactivity over a long duration following administration [20, 21]. Therefore, the use of AMS as the detection method in this study enabled patients to be discharged from the clinic after an initial 7-day confinement period, with four additional 24-h overnight clinic visits. This significantly reduced the burden on study participants compared with a more traditional ADME study approach (i.e., higher radioactivity dose, liquid scintillation counting), which would have required patients to remain confined to the clinic for a much longer period of time. This approach has also been used in a study of the drug vismodegib in healthy adults in order to reduce exposure to radioactivity for the participants and to improve the accuracy of detection over the duration of the study [24].

Ixazomib is a cytotoxic drug and, as such, this ADME study was conducted in patients with solid tumors as opposed to healthy volunteers. Accordingly, it was important to design the study in a manner that balanced the need for scientific rigor with study feasibility. For this reason, additional non-radiolabeled doses of ixazomib were administered to patients on days 14 and 21 of Part A, in order to provide them with an opportunity to derive clinical benefit. Consequently, the PK of non-radiolabeled ixazomib following the [^14^C]-ixazomib oral solution dose was not characterized beyond day 14. However, this design did not compromise the objective of characterizing mass balance, as the excretion of total radioactivity in urine and feces could be adequately characterized over 35 days using the discontinuous collection approach that preserved feasibility and minimized inconvenience for cancer patients. Importantly, authentic estimation of cumulative excretion over the 35 day period (>3 t_1/2_) was enabled by integration of the amount excreted between the discontinuous collection periods based on excretion rate versus time analyses. From a broader perspective, this method of analysis of data is of particular value for mass balance studies of long half-life drugs performed in cancer patients, as a traditional summation of amounts excreted over continuous collection intervals will not suffice to obtain authentic estimates of mass balance. Additionally, it would be practically infeasible to hold advanced cancer patients in the clinical research unit for extended periods of time over several weeks post-dose to enable continuous collection of excreta until achievement of approximately 80–90% cumulative recovery.

After administration of the [^14^C]-ixazomib oral solution, ixazomib and TRA were rapidly absorbed with median plasma T_max_ values of 0.5 h. The mean plasma AUC_0–312_ ratio of ixazomib to TRA was approximately 70%, indicating that the majority of systemic exposure to ixazomib-derived radioactivity was attributable to ixazomib. Concentrations of TRA in whole blood were higher than those observed in plasma; the mean whole blood to plasma TRA ratio for AUC_0–312_ was 10.5, suggesting preferential distribution of drug-related material into whole blood. This ratio is similar to the whole blood to plasma AUC_0–168_ ratios for ixazomib alone (day 1, 12.7; day 15, 9.86) that were observed following administration of 4 mg capsules to patients with relapsed or refractory light-chain amyloidosis [6]. This preferential distribution may be explained by the high concentration of proteasomes present in blood cells, and is supported by data from a physiologically-based PK study of bortezomib distribution in mice [25]. Of note, a similar observation has also been reported for bortezomib, another proteasome inhibitor, in which concentrations in whole blood were 3-fold higher than those observed in plasma in patients with MM [26].

The mean total recovery of TRA was 83.9%. Although less than the 90% “preferable” value specified in some regulatory guidelines [27], the total recovery value was sufficient to meet the study objective of characterizing the primary routes of excretion for ixazomib. In a retrospective analysis of human mass balance data for 27 compounds [19], only two out of seven (28%) compounds with TRA half-lives of >75 h were able to demonstrate recovery of ≥85% of TRA, supporting an inference that a recovery of ≥85% is typically achievable only for compounds with TRA half-lives ≤75 h. Although not estimable in the present study (despite sampling up to 35 days post dose), the t_1/2_ for TRA can be expected to be ≥9.5 days (i.e., no shorter than that of the parent drug). Therefore, the observed recovery for ixazomib TRA in the present study is consistent with its PK profile (i.e., long t_1/2_).

The majority of administered radioactivity (62.1%) was recovered in the urine, as compared with the feces (21.8%). The urinary recovery data suggest that at least 62% of the orally administered dose was absorbed into the systemic circulation. This value is consistent with the absolute bioavailability estimate of 58% from a population PK analysis that included data collected after both intravenous and oral administration of ixazomib [15]. Additionally, as only a small portion of the administered dose (3.23%) was recovered in the urine as unchanged ixazomib up to 168 h post dose, most of the TRA recovered in the urine is likely to be attributable to metabolites.

Patients with mild or moderate renal impairment (CrCl ≥30 mL/min) can begin ixazomib therapy at a starting dose of 4 mg [10], partly due to the observation that ixazomib exposures are similar to patients with normal renal function [15]. This observation is further supported by the minimal renal clearance of ixazomib observed in this study. In contrast, moderate and severe hepatic impairment resulted in 20% higher total systemic exposure of ixazomib, with an associated recommendation to reduce the starting dose to 3 mg in this patient population [10, 16], consistent with the greater contribution of hepatic metabolism (confirmed by this study). Of note, severe renal impairment and end-stage renal disease were associated with a 39% higher total systemic exposure compared with patients with normal renal function [17], resulting in a starting dose recommendation of 3 mg for these patients. Although the specific reasons for these observations are not yet known, they may be related to decreased metabolism of ixazomib in the setting of chronic severe renal impairment or end-stage renal disease, as has been described for other drugs [28, 29].

In this small population of patients with advanced refractory solid tumors for whom no life-prolonging treatment was available, the majority of TEAEs were mild in severity, affected the gastrointestinal system, and the most common events included diarrhea, headache, and decreased appetite, consistent with the known safety profile of ixazomib [2–9]. Although efficacy was not a formal endpoint in this study, it is notable that two patients had stable disease for 174 and 427 days, respectively, after single - agent ixazomib treatment.

In summary, ixazomib was rapidly absorbed and extensively metabolized after a single oral solution dose of 4.1 mg [^14^C]-ixazomib. Urinary excretion represented the predominant route of excretion of drug-related material, with very little unchanged ixazomib excreted in the urine.

## References

[1] Kupperman E, Lee EC, Cao Y, Bannerman B, Fitzgerald M, Berger A, Yu J, Yang Y, Hales P, Bruzzese F, Liu J, Blank J, Garcia K, Tsu C, Dick L, Fleming P, Yu L, Manfredi M, Rolfe M, Bolen J (2010). Evaluation of the proteasome inhibitor MLN9708 in preclinical models of human cancer. Cancer Res.

[2] Assouline SE, Chang J, Cheson BD, Rifkin R, Hamburg S, Reyes R, Hui AM, Yu J, Gupta N, Di BA, Shou Y, Martin P (2014). Phase 1 dose-escalation study of IV ixazomib, an investigational proteasome inhibitor, in patients with relapsed/refractory lymphoma. Blood Cancer J.

[3] Gupta N, Goh YT, Min CK, Lee JH, Kim K, Wong RS, Chim CS, Hanley MJ, Yang H, Venkatakrishnan K, Hui AM, Esseltine DL, Chng WJ (2015). Pharmacokinetics and safety of ixazomib plus lenalidomide-dexamethasone in Asian patients with relapsed/refractory myeloma: a phase 1 study. J Hematol Oncol.

[4] Kumar SK, Berdeja JG, Niesvizky R, Lonial S, Laubach JP, Hamadani M, Stewart AK, Hari P, Roy V, Vescio R, Kaufman JL, Berg D, Liao E, Di BA, Estevam J, Gupta N, Hui AM, Rajkumar V, Richardson PG (2014). Safety and tolerability of ixazomib, an oral proteasome inhibitor, in combination with lenalidomide and dexamethasone in patients with previously untreated multiple myeloma: an open-label phase 1/2 study. Lancet Oncol.

[5] Kumar SK, Bensinger WI, Zimmerman TM, Reeder CB, Berenson JR, Berg D, Hui AM, Gupta N, Di BA, Yu J, Shou Y, Niesvizky R (2014). Phase 1 study of weekly dosing with the investigational oral proteasome inhibitor ixazomib in relapsed/refractory multiple myeloma. Blood.

[6] Sanchorawala V, Palladini G, Kukreti V, Zonder JA, Cohen AD, Seldin DC, Dispenzieri A, Jaccard A, Schönland SO, Berg D, Yang H, Gupta N, Hui AM, Comenzo RL, Merlini G (2017) A phase 1/2 study of the oral proteasome inhibitor ixazomib in relapsed or refractory AL amyloidosis. Blood 130(5):597–605. 10.1182/blood-2017-03-771220 10.1182/blood-2017-03-771220 PMC6911836 28550039

[7] Moreau P, Masszi T, Grzasko N, Bahlis NJ, Hansson M, Pour L, Sandhu I, Ganly P, Baker BW, Jackson SR, Stoppa AM, Simpson DR, Gimsing P, Palumbo A, Garderet L, Cavo M, Kumar S, Touzeau C, Buadi FK, Laubach JP, Berg DT, Lin J, Di BA, Hui AM, Van D, V, Richardson PG (2016) Oral ixazomib, lenalidomide, and dexamethasone for multiple myeloma. N Engl J Med 374(17):1621–1634. 10.1056/NEJMoa1516282 10.1056/NEJMoa1516282 27119237

[8] Richardson PG, Baz R, Wang M, Jakubowiak AJ, Laubach JP, Harvey RD, Talpaz M, Berg D, Liu G, Yu J, Gupta N, Di BA, Hui AM, Lonial S (2014). Phase 1 study of twice-weekly ixazomib, an oral proteasome inhibitor, in relapsed/refractory multiple myeloma patients. Blood.

[9] Smith DC, Kalebic T, Infante JR, Siu LL, Sullivan D, Vlahovic G, Kauh JS, Gao F, Berger AJ, Tirrell S, Gupta N, Di BA, Berg D, Liu G, Lin J, Hui AM, Thompson JA (2015). Phase 1 study of ixazomib, an investigational proteasome inhibitor, in advanced non-hematologic malignancies. Investig New Drugs.

[10] United States Food and Drug Administration (2016) NINLARO (ixazomib) capsules, for oral use. United States prescribing information. https://www.accessdata.fda.gov/drugsatfda_docs/label/2016/208462s001lbl.pdf. Accessed November 2016

[11] EMA (2016) European public assessment report: Ninlaro. http://www.ema.europa.eu/docs/en_GB/document_library/EPAR_Scientific_Conclusion/human/003844/WC500217622.pdf

[12] Health Canada (2016) Summary basis of decision (SBD) for Ninlaro. https://hpr-rps.hres.ca/reg-content/summary-basis-decision-detailTwo.php?linkID=SBD00334. Accessed September 2017

[13] Therapeutic Goods Administration (2016) Australian register of therapeutic goods: Ninlaro. https://www.ebs.tga.gov.au/servlet/xmlmillr6?dbid=ebs/PublicHTML/pdfStore.nsf&docid=952408661B4A5520CA25806C003C9AB9&agid=(PrintDetailsPublic)&actionid=1. Accessed September 2017

[14] Gupta N, Hanley MJ, Venkatakrishnan K, Wang B, Sharma S, Bessudo A, Hui AM, Nemunaitis J (2016) The effect of a high-fat meal on the pharmacokinetics of ixazomib, an oral proteasome inhibitor, in patients with advanced solid tumors or lymphoma. J Clin Pharmacol 56(10):1288–1296. 10.1002/jcph.719 10.1002/jcph.719 PMC5069578 26872892

[15] Gupta N, Diderichsen PM, Hanley MJ, Berg D, van de Velde H, Harvey RD, Venkatakrishnan K (2017) Population pharmacokinetic analysis of Ixazomib, an oral proteasome inhibitor, including data from the phase III TOURMALINE-MM1 study to inform labelling. Clin Pharmacokinet. 10.1007/s40262-017-0526-4 10.1007/s40262-017-0526-4 PMC5648746 28290121

[16] Gupta N, Hanley MJ, Venkatakrishnan K, Perez R, Norris RE, Nemunaitis J, Yang H, Qian MG, Falchook G, Labotka R, Fu S (2016). Pharmacokinetics of ixazomib, an oral proteasome inhibitor, in solid tumour patients with moderate or severe hepatic impairment. Br J Clin Pharmacol.

[17] Gupta N, Hanley MJ, Harvey RD, Badros A, Lipe B, Kukreti V, Berdeja J, Yang H, Hui AM, Qian M, Zhang X, Venkatakrishnan K, Chari A (2016). A pharmacokinetics and safety phase 1/1b study of oral ixazomib in patients with multiple myeloma and severe renal impairment or end-stage renal disease requiring haemodialysis. Br J Haematol.

[18] Gupta N, Hanley MJ, Venkatakrishnan K, Bessudo A, Rasco DW, Sharma S, O'Neil BH, Wang B, Liu G, Ke A, Patel C, Rowland Yeo K, Xia C, Zhang X, Esseltine DL, Nemunaitis J (2017) Effects of strong CYP3A inhibition and induction on the pharmacokinetics of ixazomib, an oral proteasome inhibitor: results of drug-drug interaction studies in patients with advanced solid tumors or lymphoma and a physiologically based pharmacokinetic analysis. J Clin Pharmacol. 10.1002/jcph.988 10.1002/jcph.988 PMC5811830 28800141

[19] Roffey SJ, Obach RS, Gedge JI, Smith DA (2007). What is the objective of the mass balance study? A retrospective analysis of data in animal and human excretion studies employing radiolabeled drugs. Drug Metab Rev.

[20] Beumer JH, Garner RC, Cohen MB, Galbraith S, Duncan GF, Griffin T, Beijnen JH, Schellens JH (2007). Human mass balance study of the novel anticancer agent ixabepilone using accelerator mass spectrometry. Investig New Drugs.

[21] Lappin G, Garner RC (2003). Big physics, small doses: the use of AMS and PET in human microdosing of development drugs. Nat Rev Drug Discov.

[22] Ognibene TJ, Bench G, Vogel JS, Peaslee GF, Murov S (2003). A high-throughput method for the conversion of CO2 obtained from biochemical samples to graphite in septa-sealed vials for quantification of 14C via accelerator mass spectrometry. Anal Chem.

[23] Kim SH, Chuang JC, Kelly PB, Clifford AJ (2011). Carbon isotopes profiles of human whole blood, plasma, red blood cells, urine and feces for biological/biomedical 14C-accelerator mass spectrometry applications. Anal Chem.

[24] Graham RA, Lum BL, Morrison G, Chang I, Jorga K, Dean B, Shin YG, Yue Q, Mulder T, Malhi V, Xie M, Low JA, Hop CE (2011). A single dose mass balance study of the hedgehog pathway inhibitor vismodegib (GDC-0449) in humans using accelerator mass spectrometry. Drug Metab Dispos.

[25] Zhang L, Mager DE (2015). Physiologically-based pharmacokinetic modeling of target-mediated drug disposition of bortezomib in mice. J Pharmacokinet Pharmacodyn.

[26] Osawa T, Naito T, Kaneko T, Mino Y, Ohnishi K, Yamada H, Kawakami J (2014). Blood distribution of bortezomib and its kinetics in multiple myeloma patients. Clin Biochem.

[27] EMA (2012) Guideline on the investigation of drug interactions. http://www.ema.europa.eu/docs/en_GB/document_library/Scientific_guideline/2012/07/WC500129606.pdf

[28] Ladda MA, Goralski KB (2016). The effects of CKD on cytochrome P450-mediated drug metabolism. Adv Chronic Kidney Dis.

[29] Zhang Y, Zhang L, Abraham S, Apparaju S, Wu TC, Strong JM, Xiao S, Atkinson AJ, Thummel KE, Leeder JS, Lee C, Burckart GJ, Lesko LJ, Huang SM (2009). Assessment of the impact of renal impairment on systemic exposure of new molecular entities: evaluation of recent new drug applications. Clin Pharmacol Ther.

